# Re-asserting the Specialness of Health Care

**DOI:** 10.1093/jmp/jhab006

**Published:** 2021-06-09

**Authors:** Benedict Rumbold

**Affiliations:** University of Nottingham, Nottingham, UK

**Keywords:** Daniels, duty to assist, health care, health needs, special, specialness thesis about health care

## Abstract

Is health care “special”? That is, do we have moral reason to treat health care differently from how we treat other sorts of social goods? Intuitively, perhaps, we might think the proper response is “yes.” However, to date, philosophers have often struggled to justify this idea—known as the “specialness thesis about health care” or STHC. In this article, I offer a new justification of STHC, one I take to be immune from objections that have undercut other defenses. Notably, unlike previous utility- and opportunity-based theories, I argue that we can find normative justification for STHC in what I term our special duty to assist those unable to help themselves. It is this duty, I argue, that ultimately gives us reason to treat health care differently from other sorts of goods (even other goods meeting health needs) and to distribute it independently of individuals’ ability to pay.

## I. Introduction

Why fund health care through public taxation? Why not have a system whereby everyone’s access to health care is dependent solely on their ability to pay? After all, this is the way we tend to treat other kinds of goods, even other kinds of social goods.

One common response to these kinds of questions, perhaps, is to say that there is something “special” about health care—that we have certain moral obligations with respect to health care that we do not with regards to most other kinds of goods and that one of the things these obligations entail is that we ensure individuals can access health care irrespective of their possession of this or that other social good (wealth in particular).

Put this way, this kind of claim—typically referred to as “the specialness thesis about health care” or STHC—might seem fairly modest. STHC does not assert that health care is the *most important* or *chief good* (cf., Segall, 2007; Rumbold, 2017), nor that it is necessarily more important than any other good. To claim that health care is special is also not necessarily to claim that it is *uniquely* special. It may well be that a number of other goods are special for precisely the same reasons health care is or that there are other goods that are special for reasons entirely different from those that make health care special (specialness theses not being mutually exclusive). Instead, the thesis—as it is understood here—only asserts:

(i) that there are at least some moral obligations we are under in virtue of which we are required to provide health care,
(ii) that the same obligations do not require similar provision of other goods; and
(iii) that at least one of the things such obligations entail is that we distribute health care independently of individuals’ ability to pay.^1^

For all its apparent modesty, however, STHC has been met with rising scepticism by the philosophical community. For some writers, of course, we never had any reason for thinking health care *was* special to begin with.^2^ More damning, though, is that even those who once advocated STHC—most notably Norman Daniels—have now largely abandoned it. To be clear: such writers do not deny that health care *is* special. Rather, they argue that the specialness thesis is better understood as covering *all* goods meeting health needs, as opposed to just health care (Daniels, 2008, 29–30).^3^ Such a set, then, comprises goods traditionally understood as healthcare goods, which is to say, all those goods and services normally covered by a comprehensive public health service (cf. Daniels, 2008). However, it is also taken to cover any other good or service that reduces the incidence of disease and disability someone may experience over the course of their life; a set that, following recent discoveries in social epidemiology, looks like it would encompass a somewhat dizzying array of items (including goods and services relating to housing and one’s living environment; one’s working environment; transport; the labor market; agriculture and food; water and sanitation; social care; education, and so on—cf. Bambra et al., 2009).

Much of the reasoning that has led us to this position would appear sound. Certainly, insofar as we attribute any moral significance to individuals’ health needs in general, it would appear to have implications for how we distribute any good meeting health needs, rather than just healthcare goods. (Following Wilson, 2009, once we accept *this*, we might wonder whether there is much to be gained by continuing to describe such goods as “special.”) However, if the conclusion that we are meant to draw from this is that there is *no difference* between our duties with regard to the provision of *health care* and our duties with regard to the provision of *other goods meeting health needs* (defined following Daniels’ definitions above), then I think that conclusion would be mistaken. Rather, in this article, I argue that we still have certain duties with respect to the provision of health care that we do not with respect to other kinds of goods, even other goods meeting health needs. Moreover, I also attempt to show that one of the things such duties entail is that we ought to distribute health care independently of individuals’ ability to pay. In effect, that there is still a sense in which health care is “special.”

My argument here ultimately rests on analysis of the kinds of predicament faced by those who require healthcare interventions and how these affect our ordinary duties to assist. Specifically, I argue that because those experiencing what I call an “actual”—as opposed to “potential”—health need are rendered less able to help themselves, we have reasons to lend them our assistance over and above any reasons we would have otherwise. We have a “special” duty to assist. Now it is this special duty to assist that I claim justifies our treatment of health care as a “special” good.^4^

The article is structured as follows. In Section II, I begin by putting our present discussion into its proper context by exploring some of the ways in which writers have sought to justify STHC in the past and why such accounts are often taken to fail. In Section III, I then lay some of the groundwork for the present justification of STHC by distinguishing between individuals’ actual and potential health needs. In Section IV, I set out the normative significance of this distinction, explaining why we might think we have certain “special” duties to assist with regard to individuals’ actual health needs that we do not with respect to their potential health needs. In Section V, I explain how our special duty to assist those with actual health needs generates the claim that health care is special. In Section VI, I reply to some possible objections. I conclude in Section VII.

Before we get into the meat of the article, however, there are a couple of limitations to this argument it is worth highlighting. First, as intimated above, one of the ways the justification of STHC I offer here differs from those that have gone before is that rather than looking to ground STHC on, say, a principle of utility, or Rawlsian principle of fair equality of opportunity, my account justifies STHC on the basis of a moral duty to assist and, in particular, our “special” duty to assist those unable to help themselves. However, while I spend some time establishing why the fact that individuals are unable to help themselves generates a *special* duty to assist, one thing I do not do here is defend the idea that, in general, we have a duty to assist those experiencing, or at risk of, harm (primarily for reasons of space). This is a significant limitation to the present argument—principally because it leaves it vulnerable to anyone who might deny we have any such duty in the first place. However, while I think we do have a general duty to assist and take this to be a feature of a wide range of ethical theories, for any who are less sympathetic to this idea, one way to understand this article might be as something of a hypothetical: that is, assuming we have a general duty to assist those experiencing, or at risk of, some harm, do we have a reason to think that health care is special? To which I argue: yes.

Another limitation of this article is that, although, as stated in the introduction, STHC is often taken as the first step toward justifying a publicly funded health system, the extent to which the present justification of STHC entails that we ought to fund health care through public taxation is not something I discuss here. Rather, my main aim here is simply to get clear on the nature of our moral obligations with regard to the provision of health care. The further question of how we ought to fund health care, given those obligations—whether by public taxation, charitable donation, or some other mechanism—is something I leave to another time.

## II. EXISTING JUSTIFICATIONS OF THE SPECIALNESS THESIS ABOUT HEALTH CARE

To help explain the need for a new justification of STHC, and to highlight some of the possible pitfalls such a theory may encounter, it is helpful, perhaps, to revisit past defenses of STHC and consider why such theories have either failed or ballooned well beyond their original purpose.

Why, then, might we think that we have a different set of duties with regard to the provision of health care than we do with respect to other kinds of social goods? One thought we might offer here could be to point to the moral value of life itself. On this line of thinking, then, the reason we ought to treat health care as a special good is because health care saves lives and, given a prior duty to save whatever lives we can, we ought to distribute health care independently of individuals’ ability to pay. Here, though, we face an obvious problem. Not all healthcare goods—indeed, maybe not even the majority of healthcare goods—do save lives, nor are they designed to. Thus, the justification is too *narrow*; it fails to justify the specialness of even a majority of healthcare goods, justifying instead only the specialness of a small subset of such goods—namely, those by which we might save lives.

Another possible justification for STHC is offered by Lawrence Stern and, latterly, Thomas Schramme. For Stern, what makes health care different from other kinds of goods is its “indisputable” utility in mitigating the “severe misfortunes” of “death . . . pain and disability” (1983, 346). Schramme (2009) makes a similar point, writing that if we want to justify the specialness of health care, we need only look to the pain and harm of disease and disability.

However, the problem here is that, while these arguments capture a property shared by almost all healthcare goods, they fail to identify one that sufficiently differentiates healthcare goods from any other social good. After all, almost all social goods could be said to have *some* sort of effect on pain or suffering (or pleasure or well-being). As such, it seems difficult to see why we ought to mark out *health care* as especially special, given the specialness we might attribute to health care could also be attributed to a vast range of other goods. Instead of being too narrow, then, justifications like Stern’s and Schramme’s look too *wide*; they make too many social goods special along with health care.

In the late 1970s and early 1980s, another possible justification of STHC was offered by Norman Daniels, this time from fair equality of opportunity. Now, as mentioned in the introduction, in recent years Daniels has revised his position on STHC, such that he now holds that all goods meeting health needs are special, rather than simply health care. However, since both his original theory and the reasons why it failed are relevant to our present discussion, it is worth spending a brief moment considering it here.

Originally, Daniels defended health care’s specialness on the grounds of its contribution to what he termed our “normal opportunity range,” which is to say, the range of opportunities one would normally expect to have, given one’s particular society and set of talents (1981, 159). In motivating this view, Daniels started from four claims: first, that disease and disability constitute an impairment to our normal, or “species-typical,” functioning^5^; second, that such an impairment constitutes a “fundamental restriction” on our “individual opportunity” relative to our “normal opportunity range”; third, that one of the central effects of health care is to prevent or moderate those diseases and disabilities that constitute such an impairment; and fourth, that health care’s contribution in this respect is significant (1981, 159). From this, Daniels concluded that all those goods traditionally recognized as healthcare goods might be seen to share a distinctive feature: namely, their strategic importance for protecting the range of opportunities one can pursue relative to others within one’s particular society. On this basis, Daniels claimed that, insofar as we have any interest in defending equality of opportunity—Daniels himself supporting a Rawlsian principle of fair equality of opportunity (cf. Rawls, 1999)—we ought to treat health care as a special good.

At first glance, perhaps the main advantage Daniels’ theory enjoyed over its competitors was that it was neither too narrow nor too wide. By tying health care’s moral significance to its contribution to our *health*—and, in turn, health’s significance to its importance with regard to our opportunities—Daniels’ defense appeared to identify a property shared by all healthcare goods but not shared by many others.

However, by the late 1990s and early 2000s, this aspect of Daniels’ thesis came under increasing pressure; not, incidentally, from any philosophical argument, but primarily from new research in social epidemiology. Significantly, none of this research threatened Daniel’s contention that health care helps alleviate disease and disability when they occur. However, such research did seem to draw into doubt the efficacy of health care on the *incidence* of disability and disease, as experienced by any given individual over his lifetime. More important, according to such work, are health’s “social determinants”: our social and psychological environment, environment in early childhood, working environment, unemployment and job insecurity, friendships and social cohesion, social exclusion, effects of alcohol and other drugs, access to healthy food, sanitation, education, and so on.

Thus, if we assumed—as Daniels did—that what makes certain goods special was their ability to *prevent* those diseases and disabilities that constitute an impairment to our normal opportunity range, then, strictly speaking, STHC entailed that a far greater range of goods were special than simply health care. Rather, all those goods relating to health’s social determinants—goods like education, housing, and sanitation—should also be recognized as “special.”

To his great credit, Daniels was quick both to recognize the force of this objection (later termed the “social determinants of health objection,” Peter, 2001; Segall, 2007, 2010; Hurley, 2007, 328), and to amend his theory. Thus, abandoning a traditional formulation of STHC, Daniels now claims that *all* those goods that meet health needs are special. This latter set is taken to include health care but also *any* good or service that reduces the incidence of disease and disability; most notably, all those goods and services by which we might tackle the social determinants of health (Daniels, 2008, 29–30).

## III. ACTUAL HEALTH NEEDS AND POTENTIAL HEALTH NEEDS

Working from the foregoing section, one conclusion we might reasonably reach is that there is no good reason for thinking we ought to treat health care differently from other sorts of goods—or, if we are persuaded by Daniels’ revised thesis, no good reason for thinking we ought to treat health care differently from any other good meeting health needs (of which it now appears there are rather a lot). As suggested in the introduction, this appears the view of most philosophers working in this field. However, while I am inclined to agree with many of the objections people have put to past justifications of STHC, I think we would be wrong to conclude that STHC is unjustifiable. Rather, I think we do have certain obligations with regard to the provision of health care that we do not with respect to other kinds of goods, even other goods meeting health needs, and as such there is a sense in which health care is “special.”

In one way, the justification of STHC I lay out here is similar to Daniels’ in that it starts from a certain reading of the normative significance of individuals’ health needs. Like Daniels, I adopt a Boorsian conception of health as an absence of pathology, with “pathology” understood as a harmful departure from normal or “species-typical” functioning (cf. Boorse, 1975, 1997). Moreover, like Daniels (2008, 37), by “health needs” I understand deviations from normal functioning that require intervention. However, where Daniels treats all health needs as morally on a par, I want to suggest that our duties with respect to some health needs are different from our duties with respect to others. Specifically, I want to suggest that there is a distinction we can make here between (a) our duties with respect to those pathologies (requiring correction) that an individual is presently experiencing (what I call an individual’s *actual health needs*) and (b) our duties with respect to those pathologies (requiring correction) that an individual is not presently experiencing but which certain features of the present situation put at risk of experiencing in the future (what I call an individual’s *potential health needs*).

In the rest of this section, I explicate further the nature of the distinction between our actual and potential health needs. In Section IV, I then explain how our duties stand with respect to each kind of need and thus the distinction’s normative significance.

Put as it is above, the distinction between actual and potential health needs might seem fairly intuitive. That is, we appear to be able to easily distinguish between situations where we are presently experiencing a particular pathology (actual health need); those where we are *not* experiencing that pathology but nonetheless are at risk of experiencing it at some point in the future (potential health need); and, those where we are not experiencing such a pathology, nor are at risk of experiencing it at some point in the future (no health need in respect of that particular pathology). For example, at present, I do not have a broken leg. If I did have a broken leg, though, I would be experiencing an actual health need: that is, I would be experiencing pathology—a deviation from normal functioning—that requires correction. Similarly, insofar as my leg is not broken, I am not experiencing that actual health need. However, my not having a broken leg is ambiguous between two possible states of affairs. On the one hand, it may be that I do not have a broken leg, nor am I at risk of breaking my leg, in which case it seems clear I do not have a health need with respect to broken legs whatsoever. On the other hand, however, it may be that I do not have a broken leg at present, but I am at risk of breaking my leg, which is to say, that there are features of my present situation that make it possible that I may break my leg at some point in the future. It might be, for example, that I happen to work in a building with a particularly slippery set of steps leading up to the front door. In these kinds of cases, it is true that I do not have an *actual* health need but I do have a *potential* health need, that is, I am at risk of experiencing a particular pathology in the future (a broken leg), even though I may not actually be experiencing it at present.

In respect to these kinds of cases, then, the distinction between actual and potential health needs can look fairly sharp. However, there are certain situations that might give us pause. For example, how ought we to classify those who are currently healthy (i.e., whose condition at the present time *T*_1_ falls within the bounds of normal functioning) but is nevertheless in the process of falling ill (i.e., whose condition is declining over time in such a way that it is inevitable that, without intervention, it will fall outside the bounds of normal functioning)? Do they possess an “actual” health need or a “potential” one?

Another question relates to how we ought to classify *present* pathologies that put us at risk of *future* pathologies. For example, one consequence of osteogenesis imperfecta (“brittle bones”) is that it puts one at a higher risk of breaking one’s bones in the future than would otherwise be the case. Should we therefore consider osteogenesis imperfecta an actual health need, or a potential one, or both? We might ask similar questions about immunodeficiency conditions, genetic disorders, and, indeed, a host of pathologies we commonly experience.

On reflection, though, it looks like we have the resources to deal with both these issues. In response to the first set of cases, then, it seems reasonable to take an individual’s health as a matter of functioning both at a given point in time and over a certain period, with both being indexed to normal or species-typical functioning. Thus, we might think that even if a platelet count at T_1_ falls within a normal range (e.g., 150,000–450,000 platelets per microliter of blood), insofar as we can determine that the platelet count is steadily dropping and will shortly fall below the lower limit of that range, or is steadily rising and will shortly climb above the higher limit of that range, we might similarly conclude that these individuals are presently experiencing a harmful deviation from the norm (progressing toward thrombocytopenia in the former case and thrombocytosis in the latter). In turn, this would suggest that there are at least some actual health needs persons may experience by virtue of how their health is *deviating* from species-typical functioning despite the fact that, if we were to take their present health as a single time slice, nothing would indicate that their functioning has *deviated* to warrant classification as a pathology.

In response to the second case, it also seems reasonable to think that, insofar as we are currently suffering from a present pathology that puts us at risk of future pathologies, we are experiencing both an actual health need and a potential health need—or, more properly, we are experiencing an actual health need, one effect of which is that it engenders a potential health need. This might seem to blur the distinction between individuals’ actual health needs and their potential health needs. However, to further differentiate the two, it is worth emphasizing here that even if some of our potential health needs may be the result of an actual health need, not all are. There may be numerous features of our situation which put us at risk of experiencing a future pathology other than some pathology we are currently experiencing. Indeed, the current evidence around the social determinants of health would appear to suggest that a significant proportion of the health risks we face are *not* the result of our actual health needs. Rather, they are features of our physical, social, and economic situation, that is, our work and home environment, the state of the labor market in our area, our access to clean water, local agricultural policies, existing social and community networks, and so on. The slippery set of stairs outside my office, for example, being one such determinant.

We might reasonably talk, therefore, of actual health needs; potential health needs; actual health needs that engender potential health need (for example, osteogenesis imperfecta); actual health needs that do not engender potential health needs (there would appear to be few real-world examples here, though it looks like a conceptual possibility); potential health needs engendered by actual health needs (e.g., the risk of breaking one’s leg one incurs when one suffers from osteogenesis imperfecta); and potential health needs that are engendered by features of one’s situation other than one’s actual health needs (e.g., the risk of breaking one’s leg when one lives or works in an unsafe environment).

Needless to say, like Daniels’ justification of STHC, one thing this account does is place a rather large burden on what does and what does not constitute a harmful deviation from normal functioning. Aside from difficulties involved in establishing the precise point at which functioning is deviating, or has deviated, one lurking issue here is how we ought to understand a *harmful* deviation as opposed to, say, a neutral or even beneficial one. Unfortunately, there is not the space to go into these matters here. However, it is worth stressing that, even if these issues pose important questions for the philosophy of medicine, in practice, the medical community has already generated countless guidelines geared towards establishing precisely when any given condition becomes an actual health need, which is to say, a present deviation from normal functioning is sufficiently harmful to require treatment. Thus, even if we lack general answers to these kinds of questions, we do not lack for specific ones.

## IV. INDIVIDUALS’ ACTUAL AND POTENTIAL HEALTH NEEDS AND THE DUTY TO ASSIST

Say we accept that there is a meaningful distinction to be made between individuals’ actual health needs and their potential health needs. Still, why think this distinction is of any normative significance? Why not talk, as Daniels does, about individuals’ health needs in general? The reason that this distinction is important, I take it, is because there would appear to be certain obligations we are under with respect to meeting one set of health needs that do not hold with respect to the other. Specifically, in what follows I want to argue that, by virtue of the fact that those experiencing an actual health need are less able to help themselves, we have a *special duty to assist* those experiencing actual health needs, which we do not with respect to those experiencing other kinds of need, even potential health needs. Making this argument requires three things: (a) an account of our *normal* duty to assist, (b) an account detailing the distinction between *special* duties to assist and *normal* duties to assist, and (c) an account of why we might have a *special* duty to assist those experiencing an actual health need which does not also obtain with respect to those experiencing potential health needs. In this section, I set out each of these in turn.

### Our Normal Duty to Assist

As I understand it here, our normal duty to assist is an obligation to help those experiencing, or at risk of, some kind of harm. Roughly stated, we might formulate such a duty in the following way:

Duty to assist (unvarnished version): If it is in our power to prevent something bad from happening to others, or to rectify a misfortune they are suffering, we ought, morally, to do it. (cf. Singer, 1972, 231–2)

Stated thus, the duty to assist can seem appealing. Certainly, it would seem somewhat incontrovertible that the fact that others are experiencing, or are about to experience, something harmful presents us with a reason to help them, or to offer them our assistance, assuming it is within our power to do so. However, one problem with unvarnished versions of the duty to assist, such as the one set out above, is that they also look far too demanding. In fact, they are too demanding. Bad things happen to people all the time. Surely, we cannot be morally obliged to be dashing constantly this way and that, preventing anything bad from happening to anybody and rectifying bad situations wherever we find them? Thus, while the fact that others are experiencing, or are about to experience, something harmful would seem to present us with a reason to offer them our assistance, it also seems clear that there is a host of further features of the moral situation that frequently speak against this requirement, features that disable those reasons to act in a wide range of cases and thus render the duty to assist less onerous across a wide range of cases. If we are to articulate a more plausible formulation of the duty to assist, then one thing we need is a clear sense of what these kinds of features are.

One thought here might be to say that our obligation to help others is disabled if our assistance is not *necessary* to prevent something bad from happening. This is Gewirth’s view (1978, 217–8). Another view, one which suggests the obligation to be more demanding (i.e., remains active in a wider range of cases), would be to say that we are only freed of our obligation to assist when such assistance requires us to sacrifice something of comparable moral importance. This is Singer’s view (1972, 231–2). Famously, however, Singer stresses that the physical distance between us and the person who requires help does *not* constitute a reason *not* to assist them, nor does the fact that others may be in a position to offer their help as well. Thus, as he puts it,

It makes no moral difference whether the person I can help is a neighbor’s child ten yards from me or a Bengali whose name I shall never know, ten thousand miles away . . . [or whether] I am the only person who could possibly do anything and cases in which I am just one among millions in the same position. (Singer, 1972, 231–2)

Putting this matter to one side for a moment, it is also worth noting that there is another problem here. That is, assuming we can reach an agreement about when we *do* have a duty to help those experiencing or at risk of harm (i.e., when it remains active), there is also the further question of what would be the most appropriate means of *fulfilling* such duties. This might be understood as a political, as well as a moral question. That is, assuming that, we—as a political, as well as moral community—have a moral obligation to prevent bad things from happening to others, or rectify misfortunes they are suffering, we might think that how we respond to that duty is something we can tackle together, as a political community. Perhaps it would make sense, for example, for us to charge the state with fulfilling such obligations on our behalf, so that we may get on with other aspects of our lives.^6^

If we are to realize the duty to assist as a practical principle capable of governing our behavior, then, we need clarity on (a) how we are to understand the demands the duty to assist makes on us and in what circumstances and (b) what we should do about it once our moral obligations have been made clear. However, although these are important questions, I do not attempt to resolve them here.^7^ Instead, rather than considering what kinds of features of a situation might *disable* existing reasons to help those suffering, or about to suffer, some harm, I want to focus on the possibility that there may be certain features of a situation that give us additional reasons to help than would otherwise be the case, features that render our duty to assist *more* stringent than it would be otherwise, which is to say, less capable of being overridden by competing moral considerations (cf. Rumbold, 2019). In my way of putting things, cases in which we might be seen to be under a “special” duty to assist.

### Special Duties to Assist

As intimated above, as I understand it here, we have a special duty to assist when certain features of a moral situation render our normal duty to assist more stringent than it would be, were it that those features did not obtain. The thought here, then, is that we might imagine two kinds of situations in which a duty to assist might arise. First, there might be a range of cases in which the only morally relevant features of the situation are the fact that someone is experiencing, or at risk of, some kind of harm and that certain disabling features (for example, that assistance would require a sacrifice of comparable moral significance) are not present. In these kinds of cases, I take it that we have a “normal” or “standard” duty to assist.

However, I also take it that there may be other kinds of cases in which there are certain *other* morally-relevant features present—features *beyond* the fact that someone is experiencing, or at risk of, some kind of harm—that give us reason to assist the affected agent *over and above* whatever reason we had by virtue of that individual’s predicament. Now it is in these kinds of cases that I want to say we have a “special” obligation to assist. That is, we have more reason to assist than we would have had otherwise, and our duty *to* assist is more demanding on our action, which is to say, less defeasible in the face of competing considerations.^8^ The thought here, then, is that where a rival consideration, P *would* present a justification for failing to assist in situation x, where the features of the situation are such that we only have a *normal* duty to assist, the same consideration, P, would *not* present a justification for failing to assist in situation y, where the features of the situation are such that we have a *special* duty to assist.

### Our Special Duty to Assist Those with Actual Health Needs (and Not Those with Potential Health Needs)

Using the distinction above between normal and special duties to assist, what I want to argue now is that we have a *special* duty to assist with regard to those with actual health needs and that the same is not true with respect to those with potential health needs.

Why think, then, that we have a special duty to assist those with actual health needs? As indicated above, as I see it, what grounds our special duty to assist those with actual health needs is that such needs are *debilitative*, which is to say, that they directly diminish the ability of those who suffer from them to meet both those needs and certain other needs.^9^ In asserting that we have a special duty to assist those with actual health needs, it becomes necessary to prove two things: that actual health needs are debilitative and that the fact that someone else is unable to help themselves gives us a special duty to assist them. Let me deal with these in turn.

Why should one think that actual health needs are *debilitative*—that is, that they directly diminish patients’ ability to meet both these and certain other needs? To a certain extent, one might see this as necessarily following from what it means to have an actual health need in the first place. As set out in Section III, to have an actual health need is to experience a deviation from normal functioning, either in the sense that one’s normal functioning has deviated or is deviating. If, then, we take our ability to function normally as a condition of our being able to meet our needs, we might similarly take our experience of an actual health need to undercut directly our ability to meet that need, as well as certain other needs we might be experiencing.

This argument might be put under pressure in two ways. First, it might be argued that our ability to meet our needs is not always dependent on our ability to function normally. For example, we might have friends or family who are able to help us meet our needs when we are otherwise debilitated. In other cases, we may have the necessary resources to insure ourselves against future deviations from normal functioning, such that we are able to continue to meet our needs even when our functioning is impaired.

Second, one might argue that not all actual health needs—which is to say, deviations from normal functioning we are presently experiencing—are debilitating in the sense suggested above. Here we might think, for example, of various asymptomatic deviations we experience, such as high blood pressure or high cholesterol, which, while clearly actual health needs, do not seem to impede our ability to meet that need or any other need.

There are a couple of things we might say in response here. First, insofar as the examples in the first counter are cases in which individuals are fortunate enough to have the resources (financial or otherwise) to meet their needs irrespective of their state of health, it seems clear that they fail to present meaningful counter-examples to the present thesis. That is, I take it that the purpose of a specialness thesis about health care is primarily to justify why we have certain moral obligations with respect to the provision of health care. One thing the examples in the first counter do is point to occasions where these provisions may not be necessary. However, the mere fact that there might be some individuals who are fortunate enough to render our obligations in this respect superfluous—they are perfectly capable of meeting such needs themselves—is not a reason to think that we have no obligations in the first place and, more importantly, no obligations with respect to those less fortunate than they. Thus, cases in which individuals may be able to meet their needs irrespective of their ability to function normally look largely irrelevant to the argument being made here.^10^

The second counter is more challenging. To begin with, the central claim made by the counter is clearly true: not all actual health needs do seem to be debilitating in the way intimated above. Moreover, as well as the possibility of there being at least some non-debilitative actual health needs, it also seems clear that some actual health needs will be less debilitating than others. Thus, insofar as the present thesis attempts to ground the normative significance of health care on the debilitating effects of individuals’ actual health needs, these facts present an important limitation to the scope of that argument. However, there are a few things to say in mitigation here. First, the fact that some actual health needs are more debilitating than others does not undermine the logic of the current argument, it simply suggests that there is a further level of granularity at which we might assess the normative significance of different needs: not all debilitative actual health needs are necessarily on a par.^11^ Second, while it may be true that there are some entirely non-debilitating actual health needs, these look to be relatively few in number. Indeed, one thing that it may be worth emphasizing here is that, in order to qualify as an actual health need on the current conception, the deviation one experiences from normal functioning must be a harmful one. Thus, any *benign* or *neutral* asymptomatic deviation from normal functioning would fail to qualify as non-debilitating actual health needs, as they would first fail to qualify as an actual health need. This leaves, of course, an important class of harmful asymptomatic deviations from normal functions left outside the scope of the present thesis, and with respect to these, one is simply forced to concede that the present argument provides no justification for their moral significance. However, even here, it is important to note that simply because such needs are not covered by the present justification of STHC does not necessarily mean that we have no good reason to remedy them.

Accepting, therefore, that not all actual health needs are necessarily debilitating, we might still conclude that the vast majority are (to a greater or lesser extent). Still, even if we think that (many) actual health needs are debilitating, why think that this fact about them grounds a special duty to assist?

Perhaps the first thing to say is that I take it for granted that, when others are experiencing an actual health need, we at least have a *normal* duty to assist. That is, provided certain disabling conditions do not apply, the fact that others are experiencing harmful deviations from normal functioning that require intervention would appear to present us with reason enough to help them, or to offer them our assistance, assuming it is within our power to do so. The further thought here, then, is that insofar as someone is experiencing an actual health need, the fact that that need is also a *debilitating* one—it renders them less able to help themselves—gives us *additional reasons* to help them, over and above those reasons we have by virtue of the harm they are experiencing. In other words, there is a *special* duty to assist.

That individuals’ (in)ability to help themselves affects our duty to assist in this way follows, I believe, from how responsibilities for meeting needs are distributed among the field of possible responders. Here, it is worth recalling that on this precise point Singer sticks closely to a principle of equity. Thus, on his view, the duty to assist (rendered in terms of a duty to rescue) “makes no distinction between cases in which I am the only person who could possibly do anything and cases in which I am just one among millions in the same position” (Singer, 1972, 231–2). On this view, then, our responsibility to assist is neither shared between whoever can assist, nor held by some agents to a greater degree than others. Rather, it binds each possible respondent equally, each having equal reason to help (see also, Unger, 1996).^12^

I am pretty sympathetic to this view. However, even if we might agree that the duty to assist falls equally on all possible respondents *other than the agent suffering the need*, it would still seem to be the case that, insofar as any agent, P, suffers a need, x, the duty to meet x falls first and foremost on P. To suggest otherwise would seem hopelessly alienating. That is, it would seem to imply that the way my life goes is as much your responsibility as it is mine, that my life is not, in a sense, my own—or, at least, no more mine than it is yours.

If we assume, then, that insofar as any agent P suffers a need x, the duty to meet x falls first and foremost on P, we can begin to see why it would be morally relevant whether or not P is able to help himself. For where P *is* able to help himself, even though the mere fact that P has a need gives us sufficient reason to help him, we might also think that the *responsibility* for meeting x remains, first and foremost, P’s. Correspondingly, where P is *unable* to help himself, the responsibility for meeting x increasingly becomes *our* responsibility. We have additional reasons to help than would be the case otherwise, or, to put it another way, a special duty to assist.^13^

Since we have already established, then, that, in (most) cases, to experience an actual health need is to experience a condition such that one is less able to help oneself, we might similarly conclude that insofar as an individual suffers from a *debilitating* actual health need, we have a *special* duty to lend that person our assistance.

From this, then, I take it that we have certain special duties to lend our assistance to those with actual health needs (assuming no disabling factors apply). Here, though, we might ask: is the same true of our duties with respect to individuals’ *potential* health needs? To this I want to argue: no.

Before making this argument it is perhaps important to note that the fact that others are experiencing a potential health need does appear to give us an *ordinary* duty to assist. That is, provided certain disabling conditions do not apply, the fact that others are at risk of experiencing deviations from normal functioning that require intervention would appear to present us with reason enough to help them, or to offer them our assistance, assuming it is within our power to do so.

However, at the same time, individuals’ potential health needs do not appear to present us with a *special* obligation to assist—at least on the same grounds as our obligation to assist those with actual health needs. This is for the simple reason that unlike (almost all) actual health needs, potential health needs are *not* debilitating, that is, they do not directly diminish our ability to meet them or any other need. Again, the reasons for this are simple enough. Namely, when one experiences a potential health need, one does not experience any deviation in one’s normal functioning. Rather, one only experiences the risk of experiencing such deviations at some point in the future. As such, although we might think we have a ordinary duty to meet individuals’ potential health needs, that any given individual is experiencing a potential health need does not appear to ground the *same* special duty to assist that held with respect to individuals’ actual health needs.^14^

Following the arguments laid out above, then, we seem to have good reason for thinking that our duties with regard to individuals’ actual health needs and their potential health needs are nonidentical: we have at least some special duties to meet individuals’ actual health needs that we do not have with respect to their potential health needs. However, if we are to establish STHC, we still need to know why our duties with regard to individuals’ actual health needs entail that we have any particular obligations with regard to the distribution of *health care* (as opposed to any other good), and why such duties might counsel against the distribution of health care based on ability to pay. It is to these questions that I turn in the next section.

## V. THE DUTY TO MEET INDIVIDUALS’ ACTUAL HEALTH NEEDS AND HEALTH CARE

Having established that we have a special duty to meet individuals’ actual health needs, in order to defend STHC we require two further things: first, an account of why our duty to meet individuals’ actual health needs has any particular significance for the distribution of health care as opposed to any other kind of good, and second, an account of why our duties with regard to meeting individuals’ actual health needs demand that we distribute healthcare resources irrespective of individuals’ ability to pay.

The reason, I take it, that our duty to meet individuals’ actual health needs has a particular significance for the way we distribute health care, as opposed to any other good, is that those goods and services traditionally recognized as healthcare goods are also those goods best placed to remedy individuals’ actual health needs. What this relies on is the idea that when we are under a duty to assist, what assistance we give, and how we give it is not open. Rather, if the assistance we offer is to be a *proper* response, it needs to be one that we have good reason to believe will actually alleviate the need felt by its recipient. When it comes to our duty to assist with respect to individuals’ actual health needs, then the proper response is to provide the afflicted parties with whatever goods and services are most likely to arrest or mitigate any pathologies they are experiencing. Here, I contend that, in the main, these goods are those we usually classify as healthcare goods.

There may be, perhaps, certain cases of goods that we do not normally recognize as healthcare goods which give us pause on this point. Some studies, for example, have found that animal interaction can help improve depressive symptoms and bring about significant decreases in blood pressure values (Stasi et al., 2004; Le Roux and Kemp, 2009). Are we to assume, therefore, that pets ought to be classified as healthcare goods? Or, alternatively, that not all goods that meet actual health needs are special? I tend to take the former horn of this dilemma. That is, it seems fairly intuitive to me that insofar as we have good reason to think that any particular good can be used to meet an actual health need, then we ought to consider it as a healthcare good, even if it is not usually recognized as such. What this means, of course, is that the definition of what constitutes a “healthcare good” with which we started—namely, all those goods and services typically covered by a comprehensive public health service (cf. Daniels, 2008)—is no longer strictly true. Rather, under pressure from these kinds of examples, we might better understand “health care” to denote *any* good or service we know to be capable of meeting an actual health need, a set which would clearly include most goods and services typically covered by a comprehensive public health service but may also include certain other goods and services, the therapeutic value of which is not yet recognized by such systems.

More importantly, however, it is worth stressing here that one set of goods and services *not* covered by the present thesis (or the definition of health care above) is the vast majority of the goods other than health care which Daniels (and others) recognize as meeting health needs in general—including, for example, those relating to housing, sanitation, education, transport, and so on. This is because, while such goods and services might be enormously important in helping us meet individuals’ *potential* health needs (and hence, there is a sense in which they help us meet individuals’ health needs *in general*), they are not usually particularly well suited to helping us meet individuals’ *actual* health needs. Indeed, as emphasized in Section II, this has always been recognized by those advocating a greater focus on social determinants of health. Thus, as Wilkinson and Marmot emphasize, the point of such interventions is that they help prevent people from *becoming* ill, not that they are necessarily of any use when people *are* ill (2003, 7). For *those* kinds of needs—what I have been calling individuals’ *actual* health needs—what we require is health care. Insofar, then, as the present thesis defends the moral significance of all those goods that meet individuals’ actual health needs, it likewise asserts the moral significance of healthcare goods and healthcare goods only, effectively remaining silent on the moral significance (or otherwise) of any other kind of good, even those goods which we know help us in meeting individuals’ *potential* health needs. In this way, then, the present defense of STHC avoids the social determinants of health objection.

Let us assume, then, that the proper response to individuals’ actual health needs, given our special obligations to assist those unable to help themselves, is to provide health care, and health care only. Still, we might ask, why should our obligations in this respect entail that we ought to distribute health care separately from how other social goods are distributed, and wealth in particular?

What justifies this approach, I contend, is a tendency in our duty to assist to default towards equity; which is to say, that prior to any further consideration about whom we ought to assist, our duty to assist presumes that we ought to treat each of our fellow human beings as equal potential recipients of our assistance. One place we might see this, perhaps, is in Singer’s claim that the duty to assist (or rescue) makes no account of proximity or distance. As he puts it, “it makes no moral difference whether the person I can help is a neighbor’s child ten yards from me or a Bengali whose name I shall never know, ten thousand miles away.” One assumption behind Singer’s reasoning—to which I am sympathetic—is the thought that insofar as the duty to assist applies to anybody, it should generally be taken to apply to everybody. If, then, we are to identify certain agents as more deserving of our assistance than others, we need to have good reason to do so. By a similar line of thought, we might argue that, if our special obligation to meet individuals’ actual health needs applies to anyone, it applies to everyone. From here, then, if one were to argue that health care *ought* to be provided on the basis of ability to pay, one would need to show why those with a greater (or lesser!) ability to pay are more deserving of our assistance than others. Since, though, I know of no good argument in defense of this position, the default position would appear to provide health care—to fulfill our special duty to assist with respect to other’s actual health needs—independently of their possession of this or that social good. In short, our obligation is to treat health care as special.

## VI. OBJECTIONS, REPLIES, AND OPEN QUESTIONS

Before concluding, let me briefly respond to a few possible objections. One worry we may have about the present thesis, perhaps, is that, like the defense of STHC from utility, it can look *too wide*. Insofar as the present thesis grounds STHC on the fact that actual health needs tend to be debilitating, we might wonder whether the same argument could be used to defend the “specialness” of other goods meeting other kinds of debilitating needs. Our need for food, for example, would sometimes appear to undercut our ability to meet that need. Would it then follow that we ought to distribute food independently of ability to pay?

On this point, I find myself fairly willing to bite the bullet: yes, it does follow. However, let me say two things that may mitigate the force of this objection. First, as noted in the introduction, to defend STHC is not necessarily to claim that health care is *uniquely* special, that is, that it is the only good that ought to be distributed independently of ability to pay. Rather, to defend the specialness of health care, we only need to show that it is among a minority of goods that we should consider special. To say, then, that the present thesis makes a range of other goods special in the same sense that it makes health care special is not necessarily an embarrassment to the theory, so long as it does not suggest that *too* many other social goods are special in the way health care is (as, perhaps, the justification from utility does).

Second, to the extent that the present theory suggests that other goods ought to be treated as special in the same way as health care, I argue that, in most cases, such a conclusion often reflects our normal intuitions about *their* distribution as well. One’s need for food would appear a good example of this. That is, in those instances where an individual’s need for food does become debilitating—that is, where that need begins to undermine an individual’s ability to meet their needs—I think we often think it would be wrong to distribute food according to ability to pay. In this way, then, although the present theory may be wide in the sense of making more goods special than just health care, it is not *too* wide, in the sense of making more goods special than we would normally expect.

Another, opposite worry that one might have regarding the present thesis is that it is too *narrow*, that it does not make enough goods special. For example, much of what a modern health service does would usually fall under the category of “preventive” health care, or “public health.” However, insofar as the present thesis only defends the specialness of those goods meeting *actual*, as opposed to *potential*, health needs, it fails to account for the specialness of these kinds of services. Moreover, as highlighted in Section V, the present thesis also fails to account for the moral significance of any actual health needs that are nondebilitating. In this way, then, one might think that the present theory is too narrow in that it fails to account for the moral significance and “specialness” of all goods we would normally recognize as healthcare goods.

On the one hand, I am ready to accept the present theory’s limitations in this regard. However, I do not think that such narrowness necessarily presents a decisive objection in the same way it did, say, with respect to the “life-saving” justification of STHC. That is, I take it that the reason why the charge of narrowness was so potent in that case was that the theory failed to justify the specialness of many of the services we would usually recognize as health care. However, while the present thesis fails to justify the specialness of *all* goods we recognize as healthcare goods—and this is an important limitation—it is still able to account for the specialness of at least the majority of healthcare goods. In a way, then, while the present defense of STHC might be considered narrower than we would otherwise like, it does not seem so narrow as to be redundant.

Moreover, for those who are concerned, the present thesis suggests that all goods meeting potential health needs are *not* special, there are two things worth pointing out. First, simply because the present theory does not account for the specialness of various preventive healthcare services, that does not necessarily entail that such services ought to be distributed according to ability to pay, or, indeed, that they are not special for reasons other than the ones discussed here. Rather, it only means that we have no reason to treat such goods differently from any other *by the tenets of this particular theory*. Assuming, then, that other theories of justice are able to account for more egalitarian distributions where this one does not, one can still be confident that egalitarian allocations of preventative healthcare goods can be secured by those principles.

Second, in defending the specialness thesis about health care, the present theory does not suggest that meeting individuals’ actual health needs are, all things considered, more important than meeting their potential health needs—and thus that the goods by which we might meet actual health needs are more important than those meeting potential health needs. Indeed, it may be that, when we come to consider all the demands on our action, we find that in several cases we ought to meet individuals’ potential health needs before their actual health needs. Again, as noted in the introduction, to say that health care is special is not to say that it is *more important* than other goods, let alone that it is the *chief* good. Rather, it is only that we have certain moral obligations with respect to ensuring individuals’ access to health care that we do not have with respect to other social goods, and that one of the things that such obligations require is that we ensure individuals have access to health care irrespective of their possession of this or that social good—wealth in particular.^15^

Does this response rob the present theory of any argumentative force? After all, if we think that, despite everything that has been said so far, it may turn out that we ought, all things considered, to meet individuals’ potential health needs before their actual health needs, what work is done by asserting that health care is special? However, there are two things it is worth stressing here. First, the concession that, in certain circumstances, we may have good reason to meet individuals’ potential health needs before their actual health needs is not equivalent to saying that the two are morally on a par. Rather, the present thesis would still suggest that there are important normative asymmetries with respect to the two kinds of need, obligations which entail that, all other things being equal, we have more reasons to meet individuals’ actual health needs than we do their potential health needs. Acknowledging that all other things are not always equal does not undercut the importance of this finding; it only registers that the present justification of STHC is not the last word when it comes to the provision and distribution of health care.

Second, it is worth emphasizing that for those who are *not* persuaded by other theories asserting moral significance of individuals’ potential health needs (say, on the grounds of their significance with respect to opportunity), the present theory provides independent grounds for thinking that, nonetheless, we ought to treat health care as a special good. In this way, then, it might be seen to preserve the specialness of health care in the face of any skepticism about the specialness of other goods meeting health needs.

## VII. CONCLUSION

Despite its intuitive appeal, philosophers have often struggled to justify the idea that we ought to treat health care differently from other sorts of social goods. Indeed, even among those who, like Daniels, subscribe to the view that all goods that meet health needs are special, there is little support for the idea that health care has some further significance that continues to differentiate it from other goods meeting health needs. In this article, however, I have pushed back on this line of thought. I have argued that we still have good reason to treat health care as special, even over and above whatever reasons we might have to treat other goods that meet health needs as special. What justifies this treatment is a normative asymmetry between our obligations with respect to individuals’ actual health needs and our obligations with respect to individuals’ potential health needs. Specifically, by virtue of the fact that (almost all) actual health needs are debilitative, we have a special obligation to assist those experiencing them, something that is not true with respect to individuals’ potential health needs. This does not entail that we have no reason to meet individuals’ potential health needs. It does, though, suggest that our obligations with respect to the distribution of health care are different from those with respect to many other goods, even other goods by which we might meet individuals’ general health needs, and that, by virtue of such obligations, we have a duty to provide individuals’ experiencing actual health needs with health care and to distribute it independently of ability to pay—in other words, to treat it as “special.”

As flagged in the introduction to this article, one important question left open by the present analysis is what we ought to do about the fact we have this obligation, now that we recognize that we have it. As suggested in Section IV, I believe there are good reasons to think that this is a political, as well as moral question. However, my aim here has primarily been to get clear on our moral obligations with regard to health care. Insofar, then, as these sorts of questions take us beyond the scope of our present enquiry, I am content, for the moment, to leave them open.

## Footnotes

1. I take this description of the specialness thesis about health care (STHC) to be broadly congruent with how it is typically understood in the literature; notably, Segall’s definition of STHC as the claim that health care is “morally important in ways that justify distributing medical resources in isolation from the way in which other social goods, and wealth in particular, are distributed” (Segall, 2007; cf. Segall, 2010), and Daniels’ definition of STHC as the claim that health care is “morally important in ways that justify (and explain) the fact that many societies distribute health care more equally than many other social goods” (Daniels, 2001).

2. I take this group to be comprised of writers united by their denial of health care’s specialness, even if they are dissociated by their position on what wider theory of distributive justice obtains. It includes, therefore, writers who see justice as best served by free-market exchanges and deny health care’s specialness in respect to such a theory (Friedman, 1991; Narveson, 2006, 372); those who see distributive justice as constrained by duties of beneficence and deny health care’s specialness in respect to such a theory (Buchanan, 1991, 180); those who adopt a broadly Rawlsian view of distributive justice and deny health care’s specialness in respect to such a theory (Peter, 2001); those who see distributive justice as a matter of choices made from equality of resources and deny health care’s specialness in respect to such a theory (Fried, 1978, 127; Pogge, 1989, 181–96; Dworkin, 1993, 886); and those who see distributive justice as a matter of equality of opportunity for welfare or luck egalitarianism and deny health care’s specialness in respect to such a theory (Segall, 2010).

3. For other writers adopting similar positions, see, for example, Anand (2002), Sen (2002), Nussbaum (1992, 214).

4. The significance of our duty to assist those in need of medical attention has largely been ignored by the secondary literature around STHC (although it is familiar in the literature around health care elsewhere—see, for example, Jonsen, 1986, 172–4; Sheehan, 2007, 352–66). However, one place where it has been raised, perhaps, is in discussions about our “duty to care.” For example, Culyer and Pauly suggest that there may be, in effect, two demands for health care: first, those demands made by a given individual (for preventive care when well and for cure, prevention of deterioration, or reduction in the speed of deterioration of health when sick), and, second, those demands created by “the rest of society,” who have a desire to “care” for that individual and express their “sympathy” or “solidarity” with them (Culyer, 1971, 1993, 299–300; Pauly, 1971). This thought has also recently been picked up by Daniel Engster (2014), who argues that “care . . . the other half of health care . . . provides the best reason for states to continue subsidizing comprehensive health services.” Unfortunately, I think that Engster’s care-justification of STHC ultimately fails, and fails on much the same grounds that previous justifications of STHC have (Rumbold, 2017). There are, however, a number of points of congruity between the justification of STHC laid out in this paper and Engster’s, and, hence, I take them to be working toward similar ends.

5. Here Daniels draws on Christopher Boorse’s (1975) seminal work on disease as a harmful departure from normal functioning, understood in a statistical sense.

6. There are those who would be unlikely to find this idea attractive. For example, anyone subscribing to a libertarian or Nozickean conception of a minimal state—that is, one limited to defense, police, and the administration of justice—would be likely to resist the idea that we may legitimately task the state with fulfilling moral obligations like our duty to assist. Interestingly, though, the situation is not necessarily straightforward here. That is, given Nozick’s account that we are justified in employing private protective associations—and eventually the state—to uphold our rights, it is not clear that he would necessarily object to employing the same bodies to fulfill our imperfect, or unipolar duties. Perhaps the libertarian might respond that it would be entirely permissible for individuals to task private bodies—or even the state—to undertake that function if they so wish; but one thing they would never accept is the idea that the state may *compel* those who do not want to subscribe to such an arrangement to contribute in any case (e.g., through general taxation). This may well bring us to an impasse. However, it at least clarifies the grounds of the disagreement, namely, as revolving around the idea that the state may legitimately compel citizens to (a) fulfill certain kinds of ethical obligations and (b) fulfill them *through* subscription to existing state mechanisms.

7. For other work that has reflected on these kinds of issues, see, for example, Herman (2001).

8. Unfortunately, the language here can be a little distracting. That is, when we normally talk about Q’s “special obligations” to P, what we mean are those obligations Q owes to P by virtue of some special relationship between them. Here we might think, for example, of parents’ obligations to their child, or obligations arising from a contract. However, as intimated above, I take the notion of a “special duty to assist” to have a broader meaning than this. That is, as I understand it, we have a “special” duty to φ *whenever* there are certain features of a moral situation that give us additional reasons to φ other than the reasons to φ we already have. This might, therefore, include reasons engendered by virtue of some special relationship between P and Q, but it is not necessarily limited by such reasons.

9. It is perhaps worth noting here that in saying this, I do not rule out the possibility that there are other features of experiencing a health need that might also ground a special duty to assist beyond the fact that they render their sufferer less able to help themselves. For example, one might think that the fact that an actual health need *currently affects* the sufferers gives us reason to help them, over and above those cases in which the harm is simply something that the sufferer may, or may not, incur in the future—as is the case with respect to potential health needs. In this particular case, my view is that such features do not necessarily give us more (as in different) reasons to assist, but rather only augment those reasons to assist we already have; principally because whether they are merely at risk of incurring one, our reason for assisting is still the same—namely, the harm they are suffering (or will suffer). Hence, to my mind at least, the fact that an actual health need is something the sufferer is presently experiencing does not look like kind of the feature that would engender a special duty to assist. However, whether such features are capable of grounding a special duty to assist or not is largely moot at this point, since, for the purposes of my argument, my only aim is to show that there is at least one feature that *is* capable of grounding a special duty to assist: namely, the fact that, insofar as one experiences an actual health need, one is typically also rendered less able to help oneself.

10. There is, perhaps, an interesting question here about whether the mere fact that some are able to insure themselves against future needs, or to meet their needs despite their ill health, undercuts or “disables” our obligations to them. We might also wonder about the case of the ascetic, who does not consider the inability to meet certain needs as problematic. However, to the extent that these issues take us beyond the scope of the present paper, considering what, if anything, might mitigate against our special obligations to assist, I do not consider them further here.

11. It is worth noting here that something similar might be said of almost all justifications of STHC. On Daniels’ revised justification of the specialness of all those goods meeting health needs, for example, it seems clear some “general” health needs necessarily present a greater obstacle to individuals’ normal opportunity range than others.

12. For an interesting counter, see Miller (2013, 194–8).

13. For some, of course, this whole discussion might seem to get the moral situation back-to-front. For example, if we were to think, along with Gewirth, that we only have a duty to assist if our assistance is *necessary* to prevent the relevant harm, and we take individuals’ inability to help themselves as one of the conditions that would make our assistance “necessary,” we might also think that we have no duty to assist *except* when individuals are unable to help themselves. However, I remain unpersuaded by this line of argument. This is in part because one thing that this position assumes is that where individuals *are* able to help themselves, the fact that they are also in need provides us with no reason to help them whatsoever. As intimated above, though, contrary to this view, I argue that, in such situations, we still have a reason to help parties in need; it is simply that we have at least one less reason to assist them than we would have had, had they been unable to help themselves.

14. One thing that this leaves open, of course, is the possibility that we might have certain special duties to meet individuals’ potential health needs that we do not with respect to individuals’ actual health needs. However, since this article is devoted to exploring the justifiability of the claim that *health care* is special, rather than any other good or goods, I leave this possibility unexplored here.

15. See Segall (2007).
